# PAD4-Mediated Neutrophil Extracellular Trap Formation Is Not Required for Immunity against Influenza Infection

**DOI:** 10.1371/journal.pone.0022043

**Published:** 2011-07-11

**Authors:** Saskia Hemmers, John R. Teijaro, Sanja Arandjelovic, Kerri A. Mowen

**Affiliations:** 1 Department of Chemical Physiology, The Scripps Research Institute, La Jolla, California, United States of America; 2 Department of Immunology and Microbial Science, The Scripps Research Institute, La Jolla, California, United States of America; Cornell University, United States of America

## Abstract

During an inflammatory response, neutrophils migrate to the site of infection where they can kill invading pathogens by phagocytosis, secretion of anti-microbicidal mediators or the release of neutrophil extracellular traps (NETs). NETs are specialized anti-microbial structures comprised of decondensed chromatin decorated with microbicidal agents. Increased amount of NETs have been found in patients suffering from the chronic lung inflammatory disease cystic fibrosis, correlating with increased severity of pulmonary obstruction. Furthermore, acute lung inflammation during influenza A infection is characterized by a massive influx of neutrophils into the lung. The role of NETs during virus-mediated lung inflammation is unknown. Peptidylarginine deiminase 4 (PAD4)-mediated deimination of histone H3 and H4 is required for NET formation. Therefore, we generated a PAD4-deficient mouse strain that has a striking inability to form NETs. These mice were infected with influenza A/WSN, and the disease was monitored at the level of leukocytic lung infiltration, lung pathology, viral replication, weight loss and mortality. PAD4 KO fared comparable to WT mice in all the parameters tested, but they displayed slight but statistically different weight loss kinetics during infection that was not reflected in enhanced survival. Overall, we conclude that PAD4-mediated NET formation is dispensable in a mouse model of influenza A infection.

## Introduction

Neutrophils are a critical component of the innate anti-microbial immune response [1], [2]. Upon recruitment to the site of infection, neutrophils kill invading pathogens by phagocytosis, release of preformed microbicidal granules, and generation of reactive oxygen species [3], [4]. In addition, they secrete newly synthesized inflammatory mediators to recruit additional immune cells to the site of infection [5], [6]. Alternatively, neutrophils can kill extracellular pathogens by releasing neutrophil extracellular traps (NETs) [7]. NET structures are composed of decondensed chromatin decorated with anti-microbial mediators such as defensins, histones, neutrophil elastase, and myeloperoxidase [8], [9]. NET-mediated killing has been described for gram-positive and gram-negative bacteria as well as fungi. Targets include *Staphylococcus aureus*, Group A streptococci, *Salmonella enterica* and *Candida albicans* [7], [9], [10], [11].

NET formation requires chromatin decondensation [12], which is associated with histone H3 and H4 deimination by peptidylarginine deiminase 4 (PAD4) [13]. Peptidylarginine deiminases (PADs) comprise a family of five calcium-dependent enzymes (PAD1–4, PAD6) that catalyze the conversion of peptidylarginine into citrulline, a process referred to as citrullination or deimination. PAD family members differ largely by tissue distribution (for review: [14]). PAD4 is the only member of the PAD family that predominantly localizes to the nucleus [15]. It is highly expressed in monocytes and neutrophils [15], [16]. PAD4 substrates include histone H3 and H4. Histone deimination is associated with the negative regulation of transcription [17], [18]. More importantly, deiminated histone H3 and H4 tails are a hallmark of neutrophil extracellular traps (NETs), linking PAD4 activity to this innate defense mechanism [13], [19]. PAD4-mediated deimination of histone H3 is induced by an array of stimuli including TNF, fMLP, LPS and H_2_O_2_ [19]. Furthermore, TNFα stimulation of neutrophils leads to increased histone H4 deimination, which is part of the extruded chromatin that forms NETs [13]. Histone deimination by PAD4 is central to NET formation, since PAD4-deficient neutrophils have lost the capacity to form NETs *in vitro* and *in vivo* [20]. In addition, PAD4-deficient mice are highly susceptible to necrotizing fasciitis upon group A streptococcus infection, due to their lack of NET formation [20].

Recently, a study has reported that increased amounts of NETs are detrimental during chronic lung inflammation [21]. Chronic neutrophil-mediated inflammation is observed in lung diseases such as chronic obstructive pulmonary disease (COPD) and cystic fibrosis (CF) [22], [23]. High levels of NETs are found in the airway fluid of CF patients and in a mouse model of CF [21]. The amount of NETs correlates with severity of lung obstruction. NET formation in CF is dependent on CXCR2-mediated signals and pharmacological inhibition of NET formation ameliorates lung inflammation in a mouse model of cystic fibrosis [21].

Acute lung inflammation during influenza virus infection is characterized by massive infiltration of neutrophils into the lungs [24], [25]. The recruitment of neutrophils is dependent on CXCR2 [26]. The role of neutrophils during influenza infection ranges from protective to pathology-promoting. Depletion of neutrophils prior to infection with influenza A leads to increased mortality in mice, suggesting a protective role for neutrophils [27]. Further, the NET mechanism appears to be perturbed in neutrophils isolated from symptomatic, feline leukemia virus infected cats, suggesting that the state of viral infection can modulate NET formation [28]. Conversely, in IRAK-M-deficient mice, increased neutrophil influx is associated with worse disease outcome and higher mortality from influenza A infection [24]. Similarly, massive neutrophil infiltration is a contributing factor to the immunopathology associated with the cytokine storm observed upon infection with highly pathogenic influenza strains like the 1918 H1N1 ‘Spanish flu’ strain and H5N1 influenza strain [29], [30], [31]. In a different viral infection model, the intra-cerebral infection of mice with lymphocytic choriomeningitis virus (LCMV), recruitment of pathogenic neutrophils and monocytes is required for vascular leakage and lethality [32]. Neutrophils are equipped to promote inflammation in order to protect the host from the invading pathogen. Although neutrophil activation is beneficial during the early stages of infection, it is often associated with tissue destruction during later stages [1], [33]. Whether PAD4-mediated NET formation influences the acute anti-viral response has not been established.

Because NETs can be detrimental during chronic lung inflammation, the objective of the current study was to examine the role of NETs during acute lung inflammation. Influenza infection creates a highly pro-inflammatory lung environment and neutrophils that are recruited to the lungs encounter inflammatory mediators, like TNFα that can trigger NET formation. We generated a PAD4-deficient mouse strain (PAD4 KO), which was defective in NET formation. We compared influenza A-infection related morbidity and mortality in PAD4 WT and KO mice. Both mouse strains could control infection to a similar degree, suggesting that PAD4-mediated NET formation is protective immunity against influenza A infection. dispensable for

## Results

### Generation of the PAD4 knockout mouse strain

**Figure 1:**
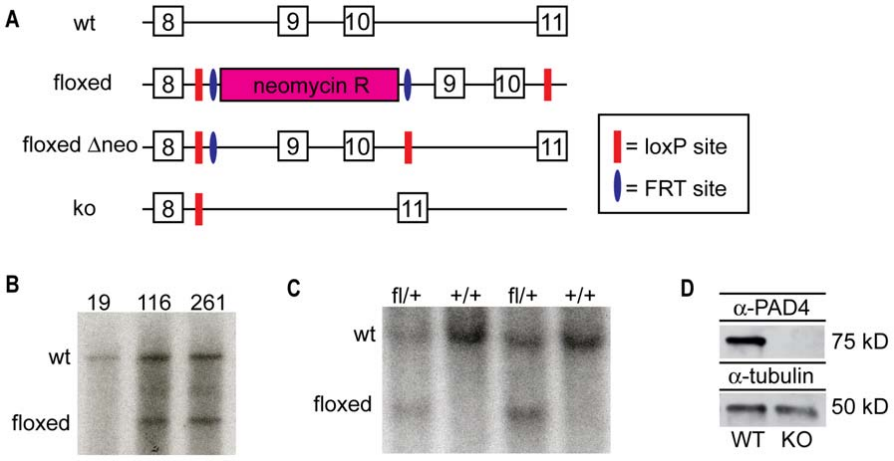
Generation and characterization of the PAD4 conditional knockout mouse strain. (A) Schematic depiction of the targeted PAD4 genomic locus. Exons are depicted as numbered boxes. (B) Genomic DNA from three ES cell clones was digested with *Hind*III and analyzed by Southern blot with a labeled 5′ probe to confirm targeting of the PAD4 locus. (C) Mouse genomic tail DNA was screened for integration of the targeting vector into the PAD4 locus by Southern blotting. Genotypes as identified by PCR are indicated on top. (D) Cells from the peritoneal cavity of PAD4 WT or PAD4 KO mice were isolated and analyzed by immunoblotting for the presence of PAD4. The membrane was reprobed with anti-tubulin to ensure equal loading between samples.

To analyze the role of PAD4-mediated NET formation during influenza infection, we generated a PAD4 deficient mouse strain. We introduced *loxP* sites into the introns flanking exons 9 and 10 of the *PAD4* gene. These exons contain aspartate 352, which is part of the active site, as well as four additional residues that are essential for Ca^2+^ binding (Q351, E353, E355, D371) [34]. The targeting vector was cloned as described in Materials and Methods. ES cells were transfected with the linearized construct and underwent G418 and ganciclovir selection (Figure 1A). Surviving clones were screened for homologous recombination by Southern blotting and two clones with proper integration of the targeting constructs were identified (Figure 1B). The ES cell clones were expanded and injected into C57BL/6 albino blastocysts. Chimeric males were intercrossed with C57BL/6 albino females to screen for germline transmission of the targeted allele (Figure 1C). Mice carrying the targeted allele (fl/+) were mated with CMV-Cre deleter mice [35] to generate PAD4+/− offspring. These mice served as the founders to establish a PAD4−/− (KO) mouse line. Animals homozygous for this deletion are viable and had no gross anatomical abnormalities (data not shown). To confirm that we had generated a PAD4 null allele, lysates of peritoneal cavity-derived cells (mainly granulocytes and monocytes) were probed with several different PAD4 antibodies, including sera generated to amino acids 2–14 of PAD4. None of the antibodies detected any signal for PAD4 in the protein lysates of PAD4 KO cells (Figure 1D and data not shown). Therefore, we have successfully generated a PAD4 conditional mouse model.

### Histone deimination is absent in neutrophils from PAD4 KO mice

**Figure 2:**
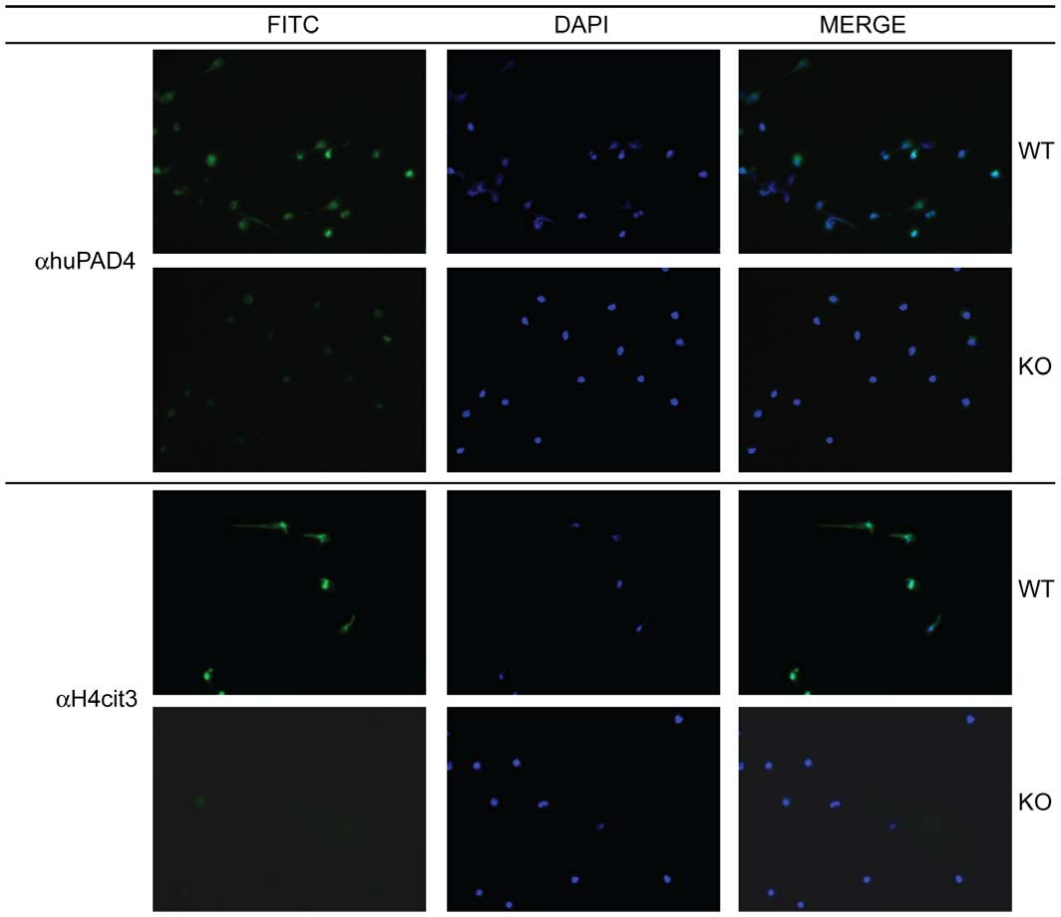
PAD4 is required for histone deimination. Casein-elicited neutrophils from PAD4 WT and KO mice were analyzed by immunofluorescence for the presence of PAD4 (α huPAD4) or deiminated histone H4 (α H4R3cit) in the FITC channel (left panel) and counterstained with DAPI to visualize DNA content (middle panel). Representative pictures from two independent experiments are shown. All pictures were taken with the 40× objective.

PAD4 catalyzes the deimination of arginine residues in histone H3 and H4 tails [17], [18]. Deiminated histones are also found in NETs released by activated neutrophils [13], [19]. A recent study described that PAD4-mediated NET formation upon inflammatory stimulation is required for efficient killing of group A *Streptococcus pyogenes* [20]. To analyze PAD4-mediated histone deimination in our mouse model, PAD4 WT and KO mice were injected with the neutrophil-eliciting agent casein. Neutrophils were harvested from the peritoneal cavity, purified, and stimulated with LPS. Harvested cells were analyzed by immuno-fluorescent microscopy. Staining with an antibody raised against human PAD4 confirmed PAD4-deficiency in neutrophils from PAD4 KO mice (Figure 2, upper panels). Neutrophils from WT mice displayed exclusively nuclear localization of PAD4. Furthermore, staining for deiminated histone H4 (Figure 2, lower panels) revealed a complete absence of deiminated histones in PAD4-deficient neutrophils. In agreement with published data [20], we also observed an absence of NET-like structures in neutrophils from PAD4 KO mice (Figure 2). Taken together, these data confirmed that deletion of exons 9 and 10 was sufficient to generate PAD4-deficient mice.

### PAD4-expressing leukocytes are recruited to the lung during influenza infection

**Figure 3:**
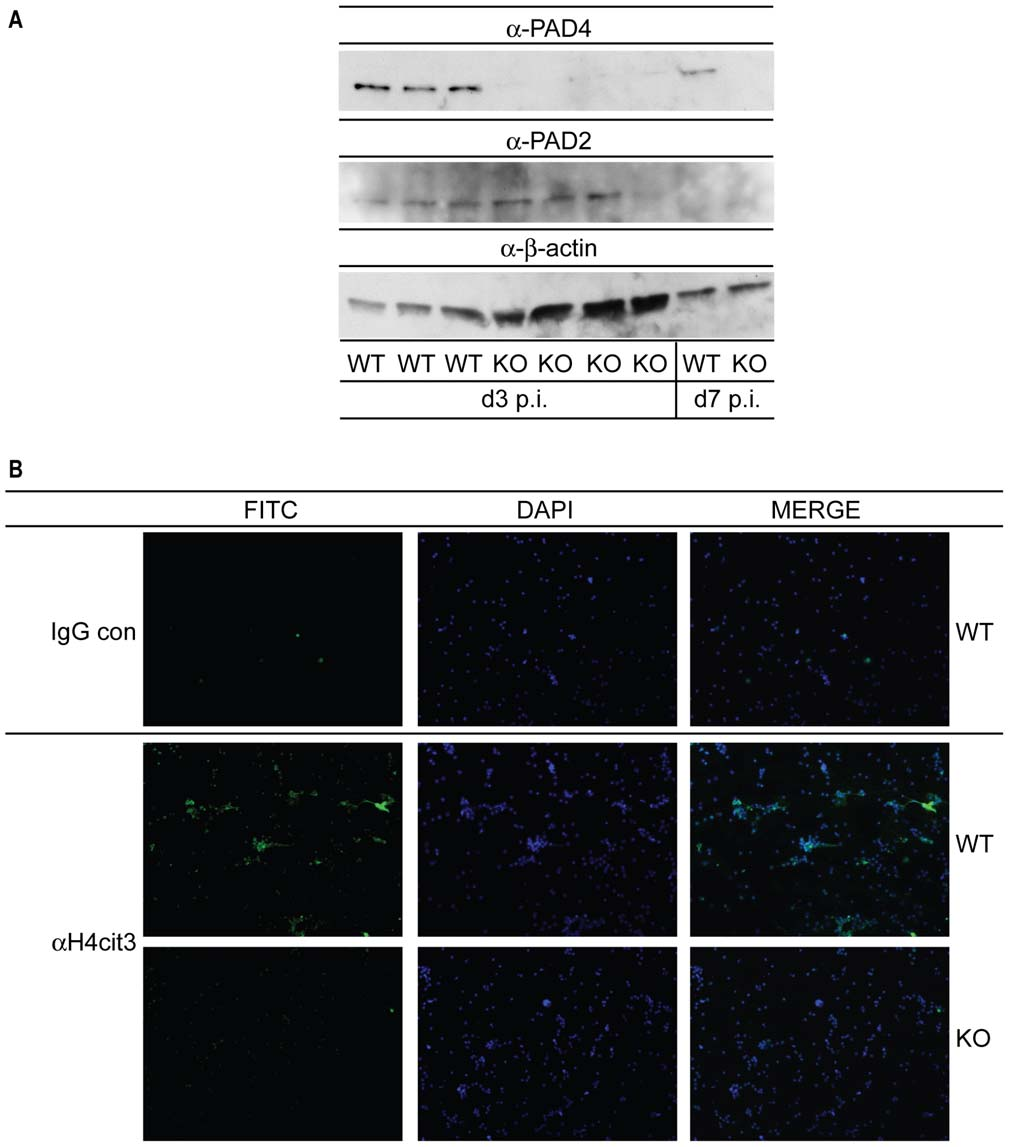
PAD4 expression and activity is detectable in the lung after influenza infection. PAD4 WT and KO mice were infected with 2000 PFU influenza A/WSN intra-tracheally (i.t.). Lungs were harvested at d3 and d7 post infection (p.i.) (5 mice/group at each timepoint) and leukocytes were isolated as described in Materials and Methods. (A) Leukocytes from individual (d3 p.i.) or pooled mice (d7 p.i.) were lysed and analyzed for PAD2 or PAD4 expression by immunoblotting. Protein loading was assessed by comparing β-actin levels in the lysates. (B) Lung leukocytes from d3 p.i. were adhered to coverslips and analyzed for deiminated histone H4 levels (α H4cit3) by immunofluorescence. Cells were counterstained with DAPI to visualize DNA. Pictures were taken with the 20× objective.

Since influenza infection induces massive inflammation in the lung [36], it was of interest to check if the inflammatory environment would induce PAD4-mediated histone deimination. Therefore, we infected both PAD4 WT and KO mice with a dose of 2×10^3^ PFU of influenza A/WSN/33/H1N1. Lungs were harvested at d3 and d7 post infection (p.i.) and leukocytes were isolated. To assess if PAD4 protein was expressed in the lung-infiltrating leukocytes, protein lysates were analyzed by immunoblotting. PAD4 expression was detected in lysates of PAD4 WT mice both at d3 and d7 p.i. and was absent in lysates from infected PAD4 KO mice (Figure 3A). Importantly, we did not observe any compensatory upregulation of PAD2, the only other PAD detected in hematopoietic cells, in PAD4 KO lysates (Figure 3A) [14]. Since PAD4 protein was detected in lung-infiltrating leukocytes, we hypothesized that the inflammatory milieu would be sufficient to induce PAD4 catalytic activity that can be measured as histone deimination [13], [19]. Indeed, deiminated histone H4 was detected in unfractionated lung leukocytes at d3 p.i. from PAD4 WT mice but was absent in isolates from PAD4 KO mice (Figure 3B and Figure S1). In summary, PAD4-expressing leukocytes were detected in the lungs of influenza A-infected mice at d3 and d7 p.i., and the inflammatory environment was sufficient to trigger PAD4-mediated histone deimination.

### PAD4-deficiency does not impact leukocyte recruitment to the lung of influenza-challenged mice

**Figure 4:**
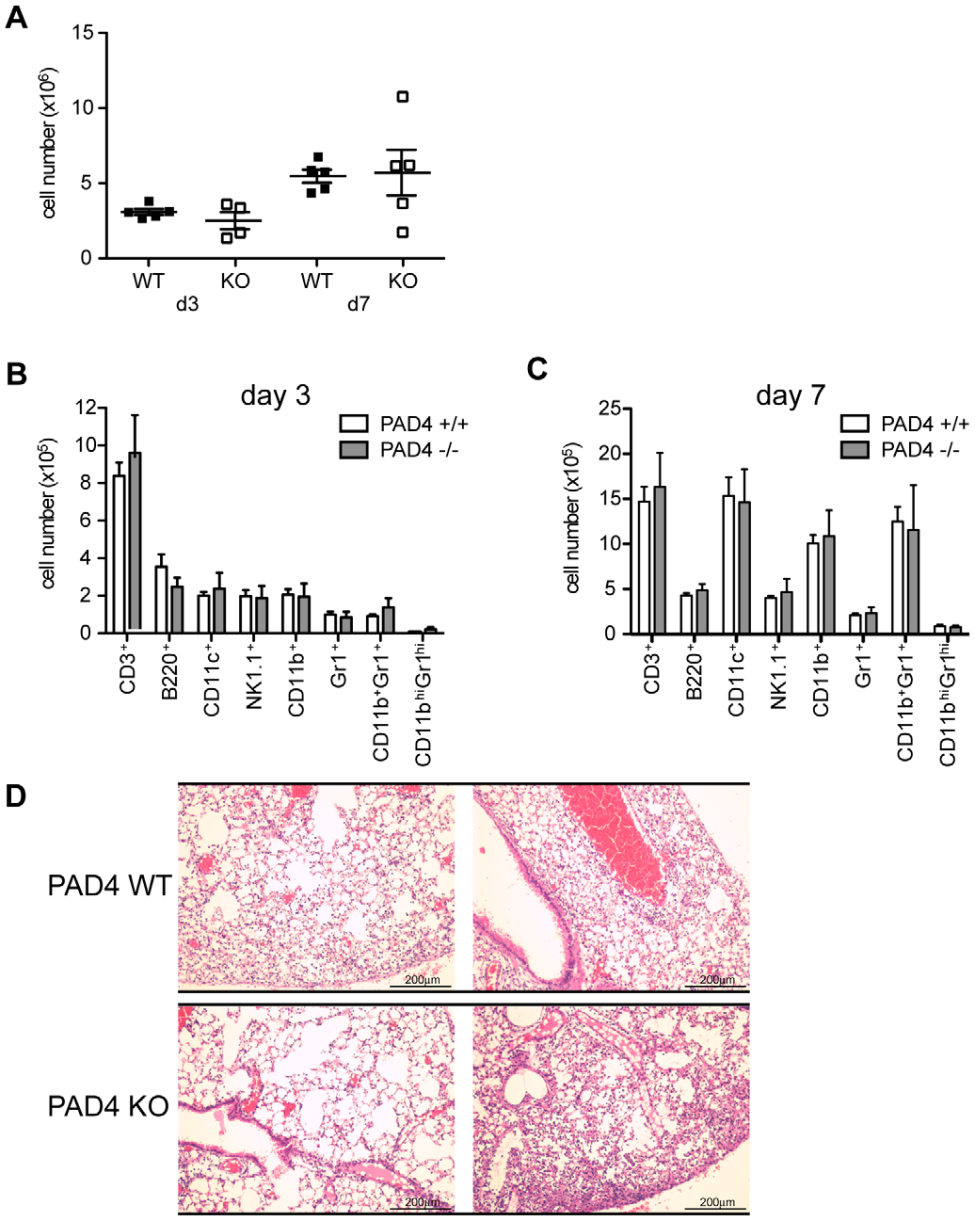
Lung leukocyte infiltration during influenza infection is comparable between PAD4 WT and KO mice. Experimental setup as described in Figure 2. (A–C) Five mice per group were analyzed at d3 or d7 post infection. Leukocytes were isolated from infected lungs. (A) Total cell numbers of lung-infiltrating leukocytes at d3 and d7 p.i. Each symbol represents an individual mouse, filled squares represent PAD4 WT, open squares depict PAD4 KO mice. (B+C) The subsets of infiltrating leukocytes were enumerated by flow cytometry both at d3 p.i. (B) and d7 p.i. (C). Gated populations are indicated at the bottom of the graph. Total numbers of infiltrating cells are shown. WT mice are depicted in white bars, KO mice as grey bars. Bars represent mean + SEM. (D) Lungs for histological examination were harvested at d8 p.i. (5 mice/group). Leukocyte infiltration was assessed on H&E stained paraffin sections. Sections from two representative mice per group are shown. The scale bar indicates 200 µm. Data is representative of two independent experiments.

PAD4 is highly expressed in neutrophils [15] and is absolutely required for NET formation [20]. Upon influenza infection, leukocytes rapidly infiltrate the infected tissue [25]. To test if PAD4 is important for neutrophil migration to the lung, we analyzed quantity and quality of infiltrating cells after influenza infection in PAD4 WT and KO mice. Both groups of infected mice showed leukocyte infiltration into the lungs both at d3 and d7 p.i. There was no difference when comparing infiltrating cell numbers between PAD4 WT or PAD4 KO mice (Figure 4A). Lung leukocytes were further analyzed by FACS analysis. There were distinct differences in cell composition between infiltrates at d3 and d7 p.i., reflected in a substantial increase in CD11c^+^ dendritic cells as well as monocyte/macrophage and granulocyte subsets (CD11b^+^ and CD11b^+^Gr1^+^) (Figure 4B and 4C). However, when comparing cell isolates from PAD4 WT mice with cells from PAD4 KO mice, the number of lung infiltrating leukocytes was similar (Figure 4B and 4C). This was also reflected in H&E-stained sections from mice at d8 p.i. Levels of inflammation were comparable between the two mouse strains as shown in Figure 4D. In summary, PAD4-deficiency has no impact on recruitment of leukocytes to the lung upon influenza A infection.

### Lung viral titers and pro-inflammatory cytokine levels are comparable between PAD4 WT and KO mice

**Figure 5:**
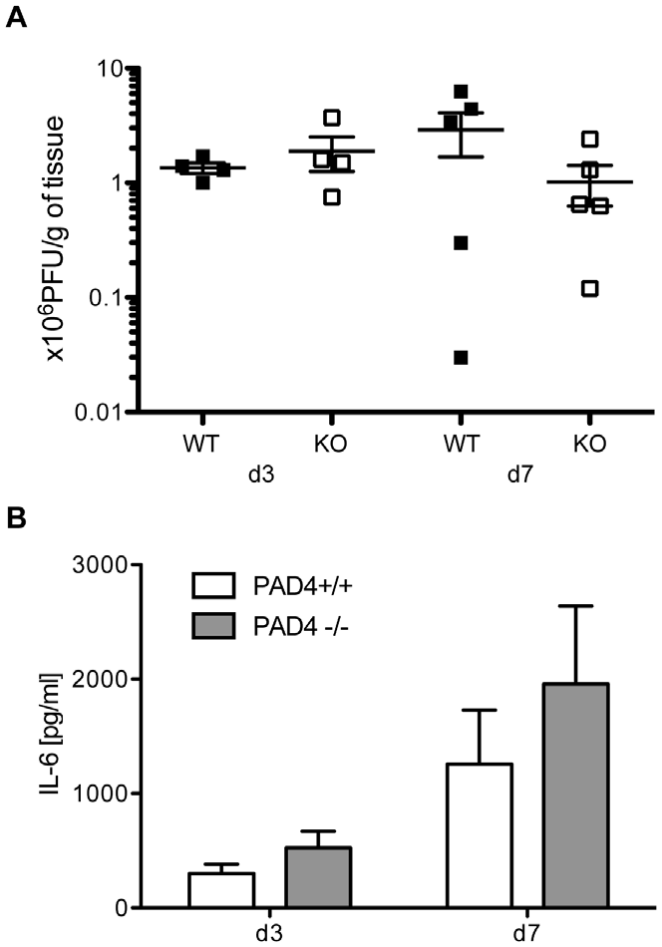
Viral titers and proinflammatory cytokine levels are similar between wildtype and PAD4-deficient mice. Experimental setup as decribed in Figure 2. Lung homogenates from five mice per group were analyzed at d3 or d7 post infection with influenza A/WSN. (A) Viral titers were assessed by a plaque-forming assay on MDCK cells. Symbols represent individual animals (filled squares = PAD4 WT; open squares = PAD4 KO). Means +/− SEM are indicated. (B) Interleukin-6 levels were analyzed by tissue homogenate ELISA. Data is adjusted to starting tissue weight. Bars represent a group of eight mice as mean + SEM (white bars = PAD4 WT; grey bars = PAD4 KO). Data are representative of two individual experiments.

PAD4-deficient neutrophils are incapable of NET formation (our observations and [20]). The importance of NETs for viral clearance has not been examined. Therefore, we set out to compare viral replication in PAD4 WT and PAD4-deficient hosts. We find that influenza virus efficiently replicated in both strains. Lung homogenates were analyzed at d3 and d7 p.i. and contained high viral titers at both time points analyzed with no significant differences between PAD4 WT and KO mice (Figure 5A). In addition, viral infection led to production of detectable levels of the pro-inflammatory cytokine IL-6, with a trend for being slightly increased in lung tissue from PAD4 KO mice, though not reaching statistical significance (Figure 5B). In conclusion, PAD4 does not restrict viral replication and does not substantially impact IL-6 production upon viral infection.

### PAD4 KO mice display decreased weight loss upon influenza infection

**Figure 6:**
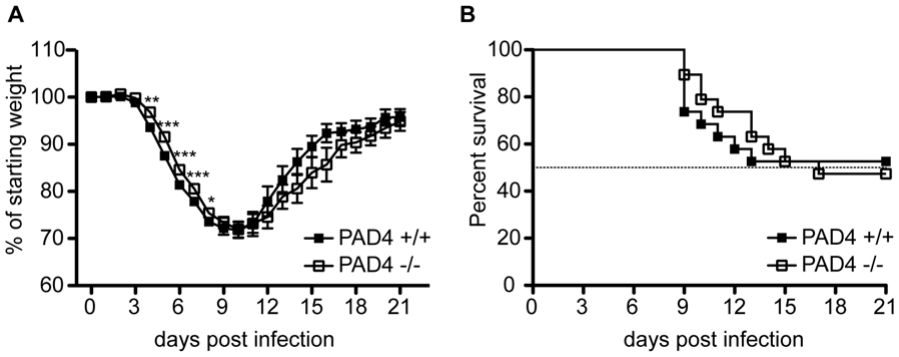
PAD4-deficient mice display a slight decrease in weight loss upon influenza infection but similar survival kinetics. Mice were infected with 2000 PFU influenza A/WSN i.t. and monitored daily for weight loss and morbidity. Data represented here includes 19 WT mice and 19 PAD4 KO mice and is the result of combining two individual experiments. (A) Mice were weighed daily and weight loss is depicted as percent of starting weight. Timepoints are plotted as mean +/− SEM, filled squares represent WT mice and open squares depict PAD KO mice. Significant differences between the groups are indictated by asteriks (p<0.05 = *; p<0.01 = **; p<0.001 = ***) (B) Survival was monitored daily for 21d p.i. The data is plotted as percent survival over time and statistical analysis was performed using the log-rank test (p = 0.9983). The dashed line indicates 50% survival. Filled squares represent WT mice, open squares depict PAD4 KO mice.

During chronic lung inflammation in cystic fibrosis, the amount of NETs in the lungs of mice and humans correlate with disease severity [21]. Since PAD4 KO mice cannot form NETs (Figure 2 and [20]), we wanted to test if this defect translated into a protective effect during influenza A infection, an acute model of lung inflammation. Therefore, PAD4 WT and KO mice were infected with influenza A/WSN and evaluated for morbidity and mortality over a period of 21 days. Animals from both experimental groups started to lose weight at d3 upon influenza infection, and they progressively lost more weight until d10 p.i. after which surviving animals began to recover and re-gain weight (Figure 6A). Interestingly, PAD4 KO mice lost significantly less weight than WT mice between d4 and d8 p.i. (Figure 6A). Both groups of mice displayed maximal weight loss at d10 p.i. with no difference between WT and KO animals and had fully regained weight by d21 p.i. (Figure 6A). The surviving PAD4 KO mice seemed to re-gain weight slightly slower than the WT mice, but the difference was not statistically significant.

The slight difference in weight loss between flu-infected PAD4 WT and KO mice had no impact on survival of mice after influenza challenge. Mice from both groups started succumbing to influenza infection at d9 p.i. (Figure 6B). PAD4 WT mice died between d9 and d13 p.i. (9/19 dead) whereas PAD4 KO mice died between d9 and d17 p.i. (10/19 dead) (Figure 6B). In conclusion, PAD4 KO mice displayed slightly different kinetics in weight change upon influenza infection, but this difference did not translate into a survival advantage.

## Discussion

Neutrophils are important mediators of innate immunity against invading pathogens. They harbor a number of different mechanisms to kill microbes ranging from phagocytosis and reactive oxygen species production to the more recently discovered NET formation [1], [2]. The importance of NETs in innate defense against gram positive and gram negative bacteria, as well as fungi, has been established [7], [8], [10]. Interestingly, NETs have been associated with enhanced pathology in patients suffering from the chronic inflammatory disease cystic fibrosis by promoting inflammation [21]. Our study focused on the role of PAD4-mediated NET formation during influenza A infection in mice.

Previous studies have firmly established that PAD4-mediated deimination of histone H3 and H4 is required for NET formation [13], [19], [20]. It has been suggested that the deimination of histones is required for proper decondensation of chromatin prior to release by the neutrophil as a part of the NET [13]. Our study examined the role of NETs during viral infection of the lung using an influenza A infection model. Therefore, we generated a novel PAD4-deficient mouse strain by crossing mice bearing a floxed allele of PAD4 with the CMV-Cre “deleter” strain (Figure 1A–D). As expected, we could not detect PAD4 protein or PAD4 activity in neutrophils from PAD4 KO mice (Figures 1D and 2). Our data is in agreement with the phenotype of one other published PAD4 conventional knockout mouse strain [20]. Since we designed the targeted allele as a conditional knockout allele, our mice will be a valuable tool for future studies, because the ‘floxed’ mice can be intercrossed with different Cre-strains to generate tissue- or cell-type specific deletion of PAD4. This is of special interest, since PAD4 dysregulation has been implicated in diseases like rheumatoid arthritis [37], [38], multiple sclerosis [39], and malignant tumors [40].

PAD4-mediated NET formation can be induced by a wide range of pro-inflammatory stimuli, including the soluble mediators TNFα, LPS, H_2_O_2_ and IL-8 [13], [19], [21]. Influenza A infection mediates lung inflammation that is characterized by influx of neutrophils into the infected lungs and high levels of inflammatory cytokines [25], [29]. Therefore, we hypothesized that neutrophils that are recruited to the highly inflammatory environment of the lung might be induced to form NETs. We tested this by isolating lung leukocytes at d3 p.i. and analyzing histone H4 deimination, a hallmark of NETs [19]. Indeed, we detected deiminated histone H4 in leukocytes isolated from PAD4 WT but not PAD4 KO mice (Figure 3B). The frequency of NET-positive cells seems low at first glance, but bulk leukocytes were analyzed and the number of CD11b^+/hi^Gr1^+/hi^ cells (monocytes and granulocytes) at d3 p.i. is only about 5–10% of total infiltrating cells (Figure 4B). For future studies, the development of antibodies that recognize deiminated histones H3 or H4 for flow cytometry should be considered. Nonetheless, this is, to our knowledge, the first report of NET formation in a mouse model of acute viral infection. These data suggest that an inflammatory environment is sufficient to trigger NET formation, even though NETs were not required for viral clearance in our model.

Influenza A infection induces extensive leukocyte infiltration into the lung [25]. PAD4-deficiency had no impact on leukocyte migration into the lung (Figure 4A–C). Similarly, viral titers were comparable between PAD4 WT and KO mice at d3 post infection (Figure 5A). However, we observed a trend towards lower viral titers at d7 p.i. and higher levels of the pro-inflammatory mediator IL-6 both at d3 and d7 p.i. when comparing PAD4 KO mice to WT controls (Figure 5A and B). Although these results did not reach statistical significance, they might explain the decrease in weight loss of PAD4 KO mice between d4 and d8 p.i. (Figure 6A). This slight but significant difference in weight loss did not translate into improved survival for PAD4 KO mice (Figure 6B). Therefore, we have to conclude that PAD4 is not essential for influenza A infection-associated immunity. Nevertheless, the observation that viral-induced inflammation is sufficient to induce PAD4-mediated NET formation is still interesting and might be more relevant during chronic viral infections. Indeed, NET formation has been shown to be associated with disease status in feline leukemia virus infected cats [28]. In a chronic setting NETs might play a role in propagating an inflamed state, similar to what has been reported for NETs in chronic lung inflammation [21]. In patients suffering from cystic fibrosis, the amount of NETs correlates with severity of pulmonary obstruction [21]. Furthermore, a recent study has shown that NETs can mediate thrombosis by stimulating platelets *in vitro*, and were also associated with thrombi in a baboon model [41]. Interestingly, common chronic infections with *Helicobacter pylori*, *Chlamydia pneumonia* and cytomegalovirus have been associated with increased thrombus formation and stroke [42]. Therefore it would be of interest to examine if any of those chronic infections are accompanied by increased levels of PAD4-mediated NET formation, possibly linking NETs to the increased risk of stroke.

Our study has provided evidence that influenza A infection can induce PAD4-mediated NET formation in the inflamed lung. By generating PAD4-deficient mice, we could test the importance of NETs for anti-viral immunity, since PAD4 is required for NET formation. During influenza A infection, PAD4-deficient mice showed similar leukocyte lung infiltration, viral titers, and IL-6 levels as compared to WT mice. Even though the weight loss kinetics were slightly different between PAD4 WT and KO mice, mortality upon influenza A infection was comparable. Therefore, PAD4-mediated NET formation is dispensable during influenza A infection.

## Materials and Methods

### Ethics Statement

All animals were handled in strict accordance with good animal practice as defined by the relevant national and/or local welfare bodies. All animal work was approved by The Scripps Research Institute Institutional Animal Care and Use Committee (protocol 09-0045).

### Mice

C57BL/6 mice were obtained from TSRI's custom breeding facility. PAD4 KO mice were generated as described below. Mice were maintained under specific pathogen free conditions in the animal facility at TSRI.

### Generation of PAD4 conditional knockout mice

The PAD4 targeting vector is based on the pRAPIDflirt vector (a gift from A. Waisman). This vector comprises a FRT-flanked PGK-neoR cassette and the HSV-thymidine kinase gene for positive and negative selection, respectively. All cloning fragments were amplified from BAC DNA that contained the whole PAD4 genomic locus (RP23-48K5). The 5′ homology arm (1.9 kb) was amplified using the following primers: 5′-TAGGATCCTCTGTGTAGCCTTGCCTGTC-3′ and 5′-TAGGATCCTATTTTGCCATTCCCAGAAAATGC -3′ and inserted into the cloning vector via *Bam*HI. The 3′ homology arm (5.3 kb) was generated with the following primers: 5′-TAGGCCGGCCTGTCCTTTGGGGGCTACACTG-3′ and 5′-TAGGCCGGCCTCACGGCTGGCTGCCTCTG-3′ and was ligated into the targeting vector via *Fse*I digestion and ligation. The region to be deleted including exons 9 and 10 was amplified with the following primers: 5′-TAGTCGACTAACTCCTGCCCTCAGCCTCT-3′ and 5′-TAGTCGACATGATGCCCAAGAGATGGAACTCT-3′ and inserted into the targeting vector between the loxP sites via *Sal*I. All cloning steps were assessed by analytical restriction digest and sequencing. The completed targeting vector was linearized with *Not*I digestion prior to electroporation of Bruce4 mouse embryonic stem (ES) cells at the Gene Targeting Core of the Scripps Research Institute.

The electroporated ES cells were subjected to positive and negative selection with G418 and ganciclovir, respectively. 300 clones were screened for homologous recombination events by standard Southern blotting techniques using *Hind*III-digested genomic ES cell DNA and a radiolabelled 5′probe.

ES cell clones that carried the desired insertion into the PAD4 genomic locus were injected into blastocysts derived from C57BL/6 albino mice and transferred into pseudopregnant females. Male chimeric offspring were mated with C57BL/6 albino females. PCR and Southern blotting of genomic tail DNA of the offspring verified germline transmission of the targeted PAD4 locus. PAD4fl/+ mice were mated with CMV-Cre deleter mice (B6.C-Tg(CMV-Cre)1Cgn/J) purchased from Jackson laboratories to generate PAD4+/− mice. Those mice were intercrossed to generate the PAD4−/− KO strain.

### Immunoblotting

Protein lysates were prepared in RIPA buffer (50 mM Tris-HCl pH 7.4, 150 mM NaCl, 1 mM EDTA, 1% triton x-100, 1% sodium deoxycholate, 0.1% SDS) including protease inhibitors. Protein concentration was determined with a BCA assay kit (Pierce). Proteins were separated by SDS gel electrophoresis and transferred onto PVDF membranes (BioRad). To block nonspecific binding, membranes were incubated in 5% (w/v) milk in TBST for 1 h at room temperature. Membranes were rinsed with TBST and incubated with the desired antibody diluted in 5% (w/v) BSA in TBST over night at 4°C. To detect the signal, membranes were incubated with for 1 h in secondary species-specific antibody diluted 1∶10000 in 5% (w/v) milk in TBST. To develop the signal, membranes were incubated with Western Lightning Plus (Perkin-Elmer) reagent and exposed to film (Denville HyBlot CL) in the dark.

Antibodies utilized in this study were: α PAD4 (2206A) purified in house (rabbit polyclonal; generated using a PAD4 peptide corresponding to amino acids 2–14) used 1∶2000; α tubulin (abcam, ab6046) (rabbit polyclonal) used 1∶3000; α β-actin (abcam, ab8226) (mouse monoclonal) used 1∶5000; α rabbit-HRP (Invitrogen, G21234) used 1∶10000; α mouse-HRP (Invitrogen, 81-6720) used 1∶10000.

### Immunofluorescence analysis of neutrophils and lung leukocytes

Neutrophils were elicited by casein-injection into the peritoneal cavity of PAD4 WT and KO mice and purified following a published protocol [43]. Lung leukocytes were isolated from PAD4 KO and control mice at d3 post influenza infection (for detailed description see below).

Elicited neutrophils were plated onto poly-L-lysine coated coverslips (0.001% in H_2_O) at a density of 1.2×10^5^ cells/coverslip in RPMI containing 0.1% BSA. Lung leukocytes were plated onto poly-L-lysine coated coverslips (0.01% in H_2_O) at a density of 3×10^5^ cells/coverslip in RPMI containing 0.1% BSA. Cells were allowed to adhere for 30 min at 37°C. The neutrophils were stimulated in RPMI with 0.1% BSA, 2 mM CaCl_2,_ and 100 ng/ml LPS for 4 h at 37°C. Cells were fixed and permeabilized for 15 min at room temperature in PBS containing 3.8% paraformaldehyde, 2% NP-40 and 1% triton x-100. Cells were rinsed with PBST (PBS with 0.1% Tween-20) and blocked for 1.5 h in PBST with 2% BSA and 5% goat serum. Primary antibodies were added (100 µl/coverslip) and incubated overnight in a humidified chamber at 4°C. The following day, coverslips were washed with PBST and incubated with secondary α rabbit-Oregon Green 488 antibody (1∶1000 in blocking solution; Invitrogen) for 2 h at room temperature. Cells were washed and counterstained with DAPI (1 mg/ml; Sigma) 1∶2000 in PBS for 15 min at room temperature. Coverslips were washed and mounted onto microscopy slides with fluorescence mounting medium from Dako.

Antibodies used in this study were α huPAD4 (rabbit polyclonal) (1∶100 in blocking solution; a generous gift from Yanming Wang) and α histone H4 cit3 (rabbit polyclonal)(1∶500 in blocking solution; Millipore 07-596).

All immunofluorescence pictures were taken on an Axiovert 100 microscope and recorded using Axiovision AC.

### Influenza virus infection

Eight to twelve week old PAD4 WT and KO mice were infected with influenza virus (A/WSN/33/H1N1) at LD_50_ (2×10^3^ PFU). Mice were anesthetized with isoflurane and 50 µl of WSN influenza virus was administered intra-tracheally (i.t.). Mice were monitored daily for weight loss and morbidity for 21 days. All infected mice were housed in biocontainment at the animal facility of TSRI.

Viral titers were determined by standard plaque-forming assays on Madine Darby Canine Kidney cells (MDCK) and are expressed as plaque-forming units (PFU) per gram tissue.

### Lung leukocyte and homogenate preparation after influenza infection

At d3 and d7 post infection with WSN influenza virus, lungs were harvested from experimental animals. The right lobes were placed on ice in Eppendorf tubes for leukocyte isolation and the left lobe was transferred into a screw cap tube for tissue homogenization.

The left lobes were minced and resuspended in 4 ml of CDTI (DMEM with 0.01% (w/v) DNaseI, 0.1% (w/v) Trypsin inhibitor-type II-S, 0.1% (w/v) Collagenase) and incubated at 37°C for 1 h. The digested lung tissue suspension was passed through a nylon mesh cell strainer and 8 ml RPMI were added. After red-blood cell lysis, the cells were resuspended in 10 ml of 35% (v/v) Percoll in RPMI and centrifuged for 10 min at 2,000 rpm. Leukocytes were resuspended in FACS buffer (PBS with 1% FBS) and cell counts were determined with a Neubauer counting chamber using Trypan Blue for dead cell exclusion. Leukocytes were further analyzed by flow cytometry, immunoblotting, and immunofluorescence.

The right lobes were weighed to determine the tissue weight. Tissue was homogenized by bead beating in 1 ml of DMEM with 0.1% BSA and 1% Penicillin/Streptomycin per tube. Cell debris was spun down for 5 min at 10,000 rpm. Supernatants were aliquotted and frozen at −80°C for virus titer analysis and proinflammatory cytokine ELISAs.

### Flow cytometry

Cells were stained with fluorochrome-conjugated antibodies and acquired using a FACS Canto flow cytometer (BD Biosciences) with a minimum acquisition of 50,000 events, and analyzed using FACS Diva (BD) and FlowJo (TreeStar Inc.) software.

The antibodies used in this study were all purchased from eBioscience and were used at the manufacturer's recommended dilutions: FITC-CD11b (ebio 11-0112), PerCP-Cy5.5-NK1.1 (ebio 45-5941), PE-B220 (ebio 12-0452), PE-Cy7-Ly6G (Gr-1) (ebio 25-5931), APC-CD11c (ebio 17-0114), APC-eFluor 780-CD3ε (ebio 47-0032).

### Lung histology

Mice were euthanized by isoflurane inhalation and lung and trachea were exposed. Lungs were injected with 4% paraformaldehyde until fully inflated and removed from the chest cavity. After paraformaldehyde fixation and paraffin embedding, lungs were sectioned and stained with hematoxylin and eosin (H&E) by the Pathology Core Facility at TSRI. Stained sections were analyzed by light microscopy on an Axiovert 100 microscope and recorded using Axiovision AC.

### Cytokine ELISA

IL-6 levels in lung homogenates of influenza virus infected mice were measured using a standard sandwich ELISA protocol. Briefly, 96 well ELISA plates were coated with 2 µg/ml IL-6 capture antibody anti-mouse IL-6 (ebioscience; MP5-20F3) diluted in carbonate binding buffer. The plates were incubated over night at 4°C. The next day, the plates were and blocked with blocking buffer (PBS with 5% BSA) for 2 h to reduce nonspecific binding. Lung homogenates and an IL-6 standard were diluted with dilution buffer in a separate low-protein binding plate. Dilutions were transferred to the blocked 96 well plate and incubated over night at 4°C. The plates were washed and 1 µg/ml biotinylated anti-mouse IL-6 antibody (ebioscience; MP5-20F3) was added to the wells. The plates were incubated for 1 h at RT, and washed again. Avidin-conjugated alkaline phosphatase (Sigma) was diluted 1∶1000 in dilution buffer and added to the wells for 30 min at RT. The detection solution was added to the wells. Color development was monitored, and the reaction was stopped by adding 2N NaOH. The absorbance was read at OD_405_ subtracting the background at OD_450_. A standard curve was generated with SoftMaxPro and only absorbances within the range were used to generate data.

### Statistical analysis

The results are expressed as mean values for individual groups +/− standard error of the mean (SEM). Statistical significance was determined by two-tailed Student's *t* test using Graphpad Prism 5 software. Kaplan-Meier survival curves were generated using Graphpad Prism 5 software, and the log-rank test was used to determine significance. *P* values of less than 0.05 were considered statistically significant.

## Supporting Information

Figure S1 **PAD4 is required for lung infiltrate histone deimination following influenza infection.** PAD4 WT and KO mice were infected with 2000 PFU influenza A/WSN intra-tracheally (i.t.), as described in Figure 3. Lung leukocytes from d3 p.i. were adhered to coverslips and analyzed for deiminated histone H4 levels (α H4cit3) by immunofluorescence. Cells were counterstained with DAPI to visualize DNA. Pictures were taken with the 20× objective. (Replicate mice from Figure 3 are depicted.)
(TIF)
