# Supplementary figures and images for: PAD4-Mediated Neutrophil Extracellular Trap Formation Is Not Required for Immunity against Influenza Infection
Source: PLoS One. 2011 Jul 11;6(7):e22043. doi: 10.1371/journal.pone.0022043 (PMC3133614; doi:10.1371/journal.pone.0022043)

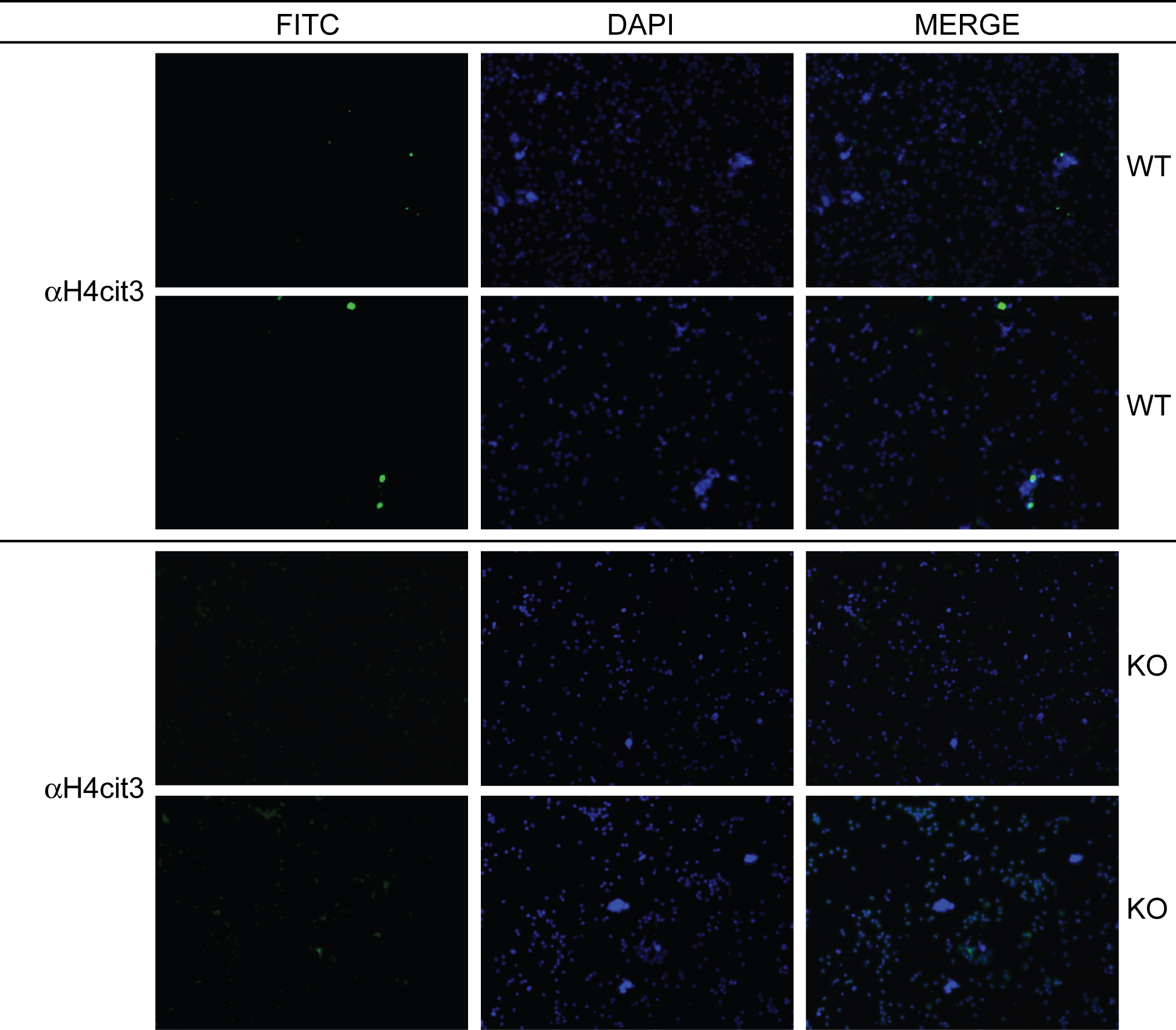

Supplement: Figure S1 — PAD4 is required for lung infiltrate histone deimination following influenza infection. PAD4 WT and KO mice were infected with 2000 PFU influenza A/WSN intra-tracheally (i.t.), as described in Figure 3. Lung leukocytes from d3 p.i. were adhered to coverslips and analyzed for deiminated histone H4 levels (α H4cit3) by immunofluorescence. Cells were counterstained with DAPI to visualize DNA. Pictures were taken with the 20× objective. (Replicate mice from Figure 3 are depicted.) (TIF) [file pone.0022043.s001.tif]
